# Measuring newborn foot length to identify small babies in need of extra care: a cross sectional hospital based study with community follow-up in Tanzania

**DOI:** 10.1186/1471-2458-10-624

**Published:** 2010-10-19

**Authors:** Tanya Marchant, Jennie Jaribu, Suzanne Penfold, Marcel Tanner, Joanna Armstrong Schellenberg

**Affiliations:** 1 Department of Global Health and Development, LSHTM, 15-17 Tavistock Place, London WC1H 9SH, UK; 2 Ifakara Health Institute, PO Box 78373, Plot 463, Kiko Avenue, Mikocheni, Dar es Salaam, Tanzania; 3 Department of Disease Control, LSHTM, Keppel St, London WC1E 7HT, UK; 4 Swiss Tropical & Public Health Institute, Socinstrasse 57, PO Box 4002 Basel, Switzerland

## Abstract

**Background:**

Neonatal mortality because of low birth weight or prematurity remains high in many developing country settings. This research aimed to estimate the sensitivity and specificity, and the positive and negative predictive values of newborn foot length to identify babies who are low birth weight or premature and in need of extra care in a rural African setting.

**Methods:**

A cross-sectional study of newborn babies in hospital, with community follow-up on the fifth day of life, was carried out between 13 July and 16 October 2009 in southern Tanzania. Foot length, birth weight and gestational age were estimated on the first day and foot length remeasured on the fifth day of life.

**Results:**

In hospital 529 babies were recruited and measured within 24 hours of birth, 183 of whom were also followed-up at home on the fifth day. Day one foot length <7 cm at birth was 75% sensitive (95%CI 36-100) and 99% specific (95%CI 97-99) to identify very small babies (birth weight <1500 grams); foot length <8 cm had sensitivity and specificity of 87% (95%CI 79-94) and 60% (95%CI 55-64) to identify those with low birth weight (<2500 grams), and 93% (95%CI 82-99) and 58% (95%CI 53-62) to identify those born premature (<37 weeks). Mean foot length on the first day was 7.8 cm (standard deviation 0.47); the mean difference between first and fifth day foot lengths was 0.1 cm (standard deviation 0.3): foot length measured on or before the fifth day of life identified more than three-quarters of babies who were born low birth weight.

**Conclusion:**

Measurement of newborn foot length for home births in resource poor settings has the potential to be used by birth attendants, community volunteers or parents as a screening tool to identify low birth weight or premature newborns in order that they can receive targeted interventions for improved survival

## Background

Despite important gains in the survival of children under five years in sub-Saharan Africa during the last twenty years, the neonatal mortality rate (NMR) remains virtually unchanged at 41 deaths within 28 days of birth for every 1000 live births. This currently represents an estimated 41% of all under five deaths [1].

The majority of these deaths occur in settings where the health system is weak and robust data is scarce. However, a recent focus on the topic has lead to some consensus about causes and actions to be taken. The three main causes of neonatal death are intra-partum related, complications due to prematurity or low birth weight, and infection [1,2]. As many as three-quarters of deaths occur in the first week of life, and up to 90% of all babies who die are born low birth weight (LBW <2500 grams) either because of prematurity or intrauterine growth restriction [2]. Community level interventions including skin-to-skin contact (also known as Kangaroo Mother Care), immediate and frequent breastfeeding, and active care seeking could reduce neonatal mortality rates by as much as 40% in high mortality settings [3-6].

There are a number of reasons why our potential to reduce the burden of neonatal death is not currently realised on a large scale [7-9]. One important road block is identification of babies at risk. Over half of all babies born in sub-Saharan Africa for example are born at home [10] and the majority of communities and families have no access to scales or other means by which to identify a baby as small, at risk, and in need of extra care.

**Figure 1:**
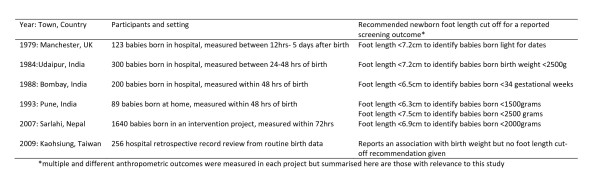
**Recommended newborn foot lengths for identification of small babies reported in peer-reviewed literature **[11-16].

Simple anthropometric alternatives to measuring birth weight have been investigated in various settings. Six separate research studies from UK, India, Nepal and Taiwan have reported on studies to investigate newborn foot length as a screening tool for small babies, showing consistent foot length cut-offs for identifying small babies across different countries (Figure 1) [11-16]. Moreover, despite having different aims and objectives, five out of six of the studies concluded that for high risk babies born at home, measuring foot length in the community may have advantages over other methods. Unlike measuring chest circumference, for example, there is a relative lack of training required, and lack of disturbance to the infant that undressing might introduce.

Here we report on the first African study of newborn foot length to identify low birth weight and premature babies and give the sensitivity and specificity estimates, and positive and negative predictive values for different foot length cut-offs. The aim was to determine the utility of using foot length as a screening tool in home delivery settings to identify low birth weight or premature babies in need of extra care. Our goal was to define a simple operational foot length cut-off that could be used to screen babies as follows: (1) babies who have an urgent need for care because they are very low birth weight (<1500 grammes); (2) babies who would benefit from additional care in the home because they have birth weight 1500-2499 grams or gestational age <37 weeks; and (3) babies who have birth weight >2499 grams and gestation age >36 weeks, and thus do not need special care because they are small.

## Methods

### Study design

We conducted a cross-sectional study of newborn babies born in hospital, with a non random subset followed up at home on the fifth day of life. A clinical officer examined babies and measured their feet on the first day, an experienced field supervisor tracked and measured the feet of babies on the fifth day; a medical doctor supervised the work. All staff were trained together on the maternity ward for one week prior to the start of data collection, each repeating foot length, birth weight and gestational age measures and comparing findings.

### Study setting and participants

The research was carried out between 13 July and 16 October 2009 within the framework of an ongoing randomised controlled trial (clinical trials identifier NCT01022788) called "Improving newborn survival in southern Tanzania" (INSIST). It is based in Lindi and Mtwara regions, a predominantly rural and poor area with high neonatal (43 per 1000 live births) and infant (76 per 1000 live births) mortality [17]. Seventy one percent of the population live within 5 kilometres of a health facility but around two-thirds of births take place at home.

During the study period, all babies born at the Mtwara Regional hospital, known as "Ligula hospital", were assessed for inclusion within 24 hours of birth and mothers asked to give informed written consent. Babies showing signs of any one of the following were excluded: severe respiratory distress, birth asphyxia, congenital deformity, birth weight <1000 grams or gestational age <28 weeks.

### Anthropometric data collection

On the first day of life, each baby recruited in hospital for whom consent was given had their right foot measured from the heel to the tip of hallux (big) toe using a stiff transparent plastic metric ruler. Birth weight was measured using digital Salter scales which were calibrated at each use with a bottle weighing 1000 grams. Gestational age was estimated by the Eregie method [18,19] which adapts the Dubowitz score [20] for African babies; in short, 6 physical characteristics of babies were examined and scored for signs of maturity (skin, eyes/ears, breast size, genitalia, mid-upper arm circumference, and head circumference).

Mothers were asked to give their home address and those estimated to live within 20 kilometres of the hospital were asked for permission for project staff to make a home visit on the fifth day of baby's life to re-measure foot length. This was then compared to the first day newborn foot length, birth weight, and gestational age estimate to explore the association up to five days after birth. Finally, project staff were asked to comment on the ease of implementation of the foot length measurement, and to report any perceptions of acceptability by parents.

### Data processing and statistical analysis

Data was entered directly into personal digital assistants at point of measurement on the first and fifth day of life, with internal consistency checks pre-programmed. Data was analysed using STATA version 10.0. The distribution of gender, birth weight, foot length and gestational age estimates for the babies measured on the first day and the fifth day subset were compared using Chi^2 ^test for heterogeneity to look for evidence of difference between the two samples that could introduce bias. We defined three binary variables to describe outcomes: (1)less than 1500 grams (very low birth weight), (2)less than 2500 grams (low birth weight) and (3)less than 37 weeks gestation (premature). Babies with these outcomes are referred to as 'small' in this context.

The absolute difference in foot length measured on the first and the fifth day for individual babies was calculated. Non-parametric receiver operating characteristics (ROC) analysis was conducted on first and fifth day foot lengths for the three outcome variables. To generate a simple operational foot length cut-off for babies with low birth weight or prematurity, a range of foot lengths was then defined as those that approximated 80% sensitivity and 80% specificity across the three outcomes. Sensitivity, specificity, positive and negative predictive values were calculated for these foot lengths.

### Ethical approval

The study was approved by the Institutional Review Board of Ifakara Health Institute, Tanzania, the Medical Research Coordinating Committee, Tanzania and the London School of Hygiene and Tropical Medicine, UK. Written, informed consent was obtained from the caregiver of each infant in the study.

## Results

### Study population

**Figure 2:**
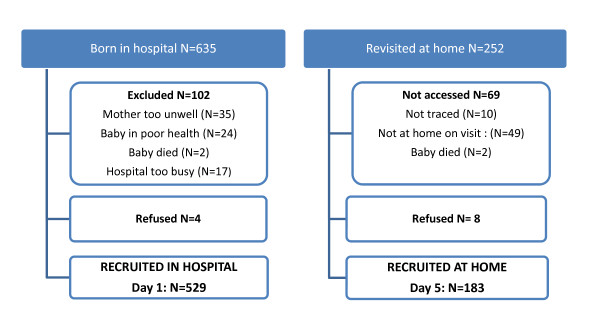
**Newborn recruitment flow chart**. Showing number of babies born in hospital during the study period who were recruited on the first day of life, and number of these babies who lived within 20 kilometres of hospital and who were visited at home on the fifth day of life.

During the study period, 635 babies were born at Ligula Hospital of whom 529 (83%) were recruited to the foot length project and had their feet measured on the first day of life. According to maternal reports, 257 lived within 20 km of the hospital and of these 183 (71%) had their feet measured again on the fifth day of life (Figure 2). The mean birth weight was 2.9 grams (SD 0.4), and mean gestational age was 39.5 weeks (SD 2.4). The two samples were similar with respect to gender, birth weight, newborn foot length and prematurity (χ^2 ^p > 0.05, table 1). Of the 529 babies, eight (2%) were born very low birth weight (<1500 grams); 78 (15%) were born low birth weight (<2500 grams); 44 (8%) were born premature <37 weeks. Amongst the premature babies, seven (16%) had birth weight <1500 grams and 37 (84%) had birth weight between 1500-2500 grams.

**Table 1:** Characteristics of newborns measured on the first day only and for the sub-sample of babies also measured on the fifth day

	All babies N = 529		First day only N = 346		First and fifth day N = 183		χ^2 4 ^p-value
Gender	n	%	n	%	n	%	

Male	283	52	188	54	95	52	

Female	246	48	158	46	88	48	0.5

Birth weight^1^							

Very LBW (<1500 g)	8	2	7	2	1	0.5	0.1

LBW (<2500 g)	78	15	55	16	23	12	0.2

Foot length^2^							

Very short feet (<7 cm)	14	3	12	3	2	1	0.1

Short feet (<8 cm)	275	52	178	51	97	53	0.7

Gestation weeks^3^							

Premature (<37 weeks)	44	8	30	9	14	8	0.6

^1^Birth weight measured within 24 hours using digital Salter scales. Mean birth weight for all babies was 2922 grams (95% confidence interval 2880-2964).

^2^Foot length measured within 24 hours of birth using a hard ruler. Mean foot length for all babies was 7.8 cm (95% confidence interval 7.8-7.9)

^3^Gestation age in weeks estimated using the Eregie method [18].

^4 ^χ^2 ^test to look for evidence of difference between the first day only and the first and fifth day subset of newborns.

The mean foot length of the 529 babies measured on the first day of life was 7.8 cm (standard deviation 0.4), and the mean foot length of the 182 babies measured on the fifth day was 8.1 cm (SD 0.3). On average, each baby's foot was 0.2 cm (SD 0.3) longer on the fifth day than on the first day.

### Sensitivity, specificity, positive and negative predictive values of foot length

**Figure 3:**
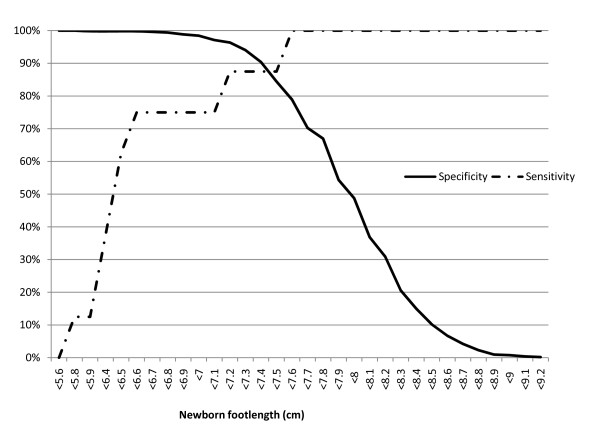
**Sensitivity and specificity of newborn foot length to predict very low birth weight (<1500 grams)**. Foot length measured on first day of life with a hard plastic transparent ruler.

**Figure 4:**
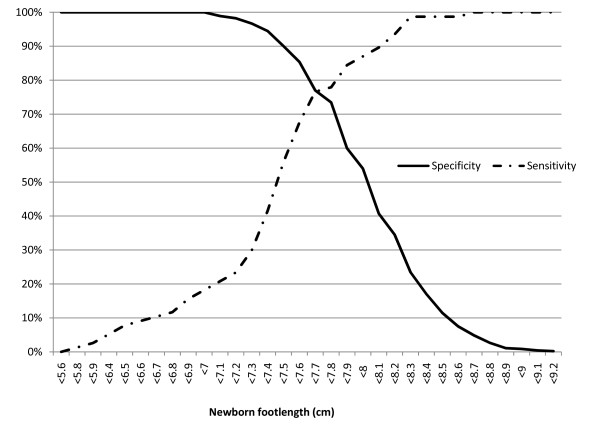
**Sensitivity and specificity of newborn foot length to predict low birth weight (<2500 grams)**. Foot length measured on first day of life with a hard plastic transparent ruler.

**Figure 5:**
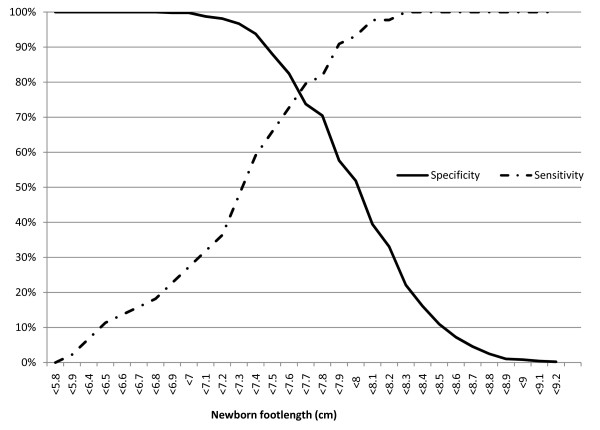
**Sensitivity and specificity of newborn foot length to predict prematurity (<37 weeks gestation)**. Foot length measured on first day of life with a hard plastic transparent ruler.

Sensitivity and specificity estimates for different foot lengths measured on the first day to identify small babies are shown in figures 3, 4 and 5 in the form of non-parametric receiver operating characteristics (ROC) curves. Assuming an 80% cut-off for both sensitivity and specificity to be desirable, we observe that this is achieved for very low birth weight (<1500 g) at foot lengths <7.2 cm and <7.5 cm respectively (figure 3), for low birth weight (<2500 g) at foot lengths <7.9 cm and <7.6 cm (figure 4), and for prematurity (<37 weeks) at foot lengths <7.7 cm and <7.5 cm (figure 5).

Using this range of foot length estimates as a guide, we show in table 2 the sensitivity and specificity for two simple and potentially operational foot length cut-offs: <7.0 cm to identify very low birth weight babies <1500 g who need urgent care and <8.0 cm to identify other small babies (low birth weight <2500 g or gestational age <37 weeks).

**Table 2:** Sensitivity, specificity, positive and negative predictive values of short foot length to identify small baby outcomes^**1 **^in southern Tanzania

First Day of life		Sensitivity (95%CI)	Specificity (95% CI)	Positive predictive value %	Negative predictive value %
*Foot length*	*Outcome*				
				
<7 cm	very LBW (<1500 g)	75% (36-100)	99% (97-99)	43	99

<8 cm	LBW (<2500 g)	87% (79-94)	60% (55-64)	24	96

<8 cm	Premature (<37 wks)	93% (82-99)	58% (53-62)	15	99

**Fifth Day of life**^**2**^					

*Foot length*	*Outcome*				
				
<8 cm	LBW (<2500 g)	77% (74-86)	74% (51-88)	29	96

<8 cm	Premature (<37 wks)	79% (73-85)	72% (44-98)	19	98

^**1 **^Small baby outcomes are very low birth weight (<1500 g), low birth weight (<2500 g), and premature (<37 weeks gestation)

^**2**^number of very low birth weight babies in fifth day subset was too small to include in analysis

Fourteen newborns (3%) had a foot length <7 cm, and 275 (52%) a foot length of <8 cm on the day of birth. Of the eight babies with birth weight <1500 g, six had foot length <7 cm and all had feet shorter than 8 cm. Of the 77 babies with birth weight <2500 g, 14 had foot length <7 cm and 67 had feet shorter than 8 cm. Of the 44 premature babies, 12 had foot length <7 cm and 41 had feet shorter than 8 cm.

Newborn foot length of <7 cm had sensitivity and specificity of 75% (95% confidence interval 36-100) and 99% (95% confidence interval 97-99) respectively to identify birth weight <1500 grams, foot length of 8 cm had sensitivity and specificity of 87% (95% confidence interval 79-94) and 60% (95% confidence interval 55-64) respectively to identify birth weight <2500 grams, and foot length of 8 cm had sensitivity and specificity of 93% (95% confidence interval 82-99) and 58% (95% confidence interval 53-62) to identify premature babies.

On day five, foot length of 8 cm had sensitivity and specificity of 77% (95% confidence interval 74-86) and 74% (95% confidence interval 51-88) respectively to identify low birth weight babies, and foot length of 8 cm had sensitivity and specificity of 79% (95% confidence interval 73-85) and 72% (95% confidence interval 44-98) to identify premature babies (table 2).

The positive predictive values (PPV) for these foot length cut-offs were low, being highest at 43% for the 7 cm cut-off to predict very low birth weight. The negative predictive values (NPV) were high at 96% for low birth weight and 99% for very low birth weight (table 2).

### Qualitative observations

Project staff reported that using the ruler to measure feet was simple to learn and to explain to others: overall, the approach was easily understood by parents. The exceptions were the four mothers who refused in hospital who reported a fear of association with measuring baby length for coffins, and the eight fathers who refused at the home visit because they had not been present for, or informed about, the participation in hospital on the first day.

## Discussion

Measuring newborn foot length in Tanzania using a simple transparent plastic ruler can identify babies needing extra care because they are low birth weight or premature. A foot length of less than 7 cm at birth was75% sensitive and 99% specific to identify very low birth weight babies (<1500 grams) and foot length less than 8 cm had sensitivity and specificity of 87% and 60% to identify those with low birth weight (<2500 grams), or 93% and 58% to identify those born premature (<37 weeks). The average newborn foot length increased by only 0.2 cm by the fifth day of life, and foot length measured on day five identified more than three-quarters of babies who were born small. The foot length cut-offs of 7 and 8 cm defined in this African setting were very similar to that previously recommended to identify small babies in Europe (7.2 cm) [11], and in Asia (between 6.3 cm and 7.2 cm) [12-15].

### Strengths and limitations of the study

This was a cross sectional hospital based study of babies at birth with a community follow-up on the fifth day of life: findings suggest that measuring newborn foot length even days after birth could be useful to identify babies needing extra care. We defined 'small babies' both by birth weight and by gestational age but had a small sample size for analysis of the relatively rare occurrence of very low birth weight (<1500 g). Although the hospital based recruitment was not representative of the population level, the prevalence of low birth weight (<2500 g, 15%) was consistent with previous findings [21]. Very little data exists at the population level with which to calculate prematurity rates in sub-Saharan Africa. Our estimate of 8% was lower than the 20% estimate reported previously from Malawi [22] with the implication that we may have underestimated the specificity for prematurity.

### Public health implications

The utility of an anthropometric surrogate to identify small babies in the community will largely be determined by the implementing environment [14]. The Lives Saved Tool (LiST) [23] estimates that in a high mortality setting such as Tanzania, 76% of neonatal deaths could be prevented if mothers and newborns were connected to comprehensive obstetric care, neonatal resuscitation and management of encephalopathy [9]. However, the majority of newborns live in environments characterised by gaps in coverage of essential interventions, in quality of health services, and in equity [24].

In 2009, WHO and UNICEF released a joint statement promoting community volunteer programmes to make home visits to newborns as a strategy to improve survival, especially where access to facility-based skilled care is limited [25]. In Tanzania, and other African and Asian countries, there is growing political support for such programmes, but some reluctance for volunteers to carry weighing scales, because of fears about cost, maintenance, and sustainability. In these settings, the use of a simple, cheap and reliable foot length tool to screen for low birth weight or premature babies could greatly strengthen the potential health impact of community volunteer programmes.

**Figure 6:**
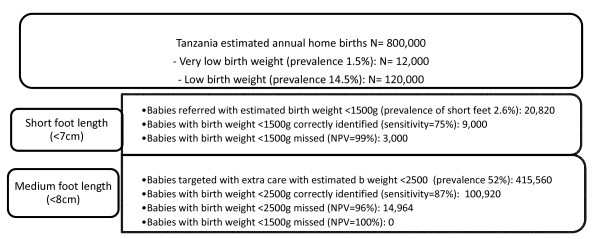
**Public health implications of community foot length screening for all home births in Tanzania**. Prevalence of low birth weight and short foot length estimates taken from this study.

In figure 6 we consider foot length screening in the context of a hypothetical community volunteer programme for the estimated annual 800,000 home births in Tanzania, of whom 12,000 babies could be expected to be born with very low birth weight and 120,000 with low birth weight. If volunteers used a screening cut-off of 7 cm, 20,820 newborns would be targeted with advice about urgent medical care for very low birth weight, 9,000 of whom would be correctly targeted; 3000 very low birth weight babies would be missed. If a screening cut-off of 8 cm was applied, 415,560 newborns (half the home birth population) would be targeted with advice about additional care for low birth weight babies in the home, 100,920 of whom would be correctly identified. Using these cut-offs in combination, fifteen thousand low birth weight babies across the country would be missed, but all very low birth weight babies would be captured.

### Further research

Two previous studies have reported high inter- and intra-observer agreement of foot length when measured by trained medical staff [11,12]. However, the reliability of foot length when measured by birth attendants, community volunteers and the mothers themselves is not yet known. We are unaware of any research or programme using foot length as a proxy for birth weight or prematurity on a large scale. This home screening tool has unknown, but promising, potential for mortality impact when integrated with home-based interventions for moderately low birth weight babies including skin-to-skin care, exclusive breastfeeding, and recognition and referral of danger signs [3-6,8].

## Conclusions

In resource poor settings where neonatal mortality remains disproportionately high, there are many missed opportunities to provide extra care to premature or low birth weight babies born at home. This study has shown that the simple and inexpensive measurement of newborn foot length can be used to screen for low birth weight and prematurity, with great potential for impact on newborn survival.

## Competing interests

The authors declare that they have no competing interests.

## Authors' contributions

TM and JAS were responsible for the study concept and design. Statistical analysis and interpretation was conducted by TM, JJ, SP, MT and JAS. TM wrote the first draft, TM, JAS, SP, JJ and MT revised the paper and contributed to discussion. All authors read and approved the final manuscript. TM acts as guarantor for the study.

## Pre-publication history

The pre-publication history for this paper can be accessed here:

http://www.biomedcentral.com/1471-2458/10/624/prepub
